# Interactions of Bromocarbazoles with Human Serum Albumin Using Spectroscopic Methods

**DOI:** 10.3390/molecules23123120

**Published:** 2018-11-28

**Authors:** Xiaodan Yan, Dongjie Yuan, Dandan Pan

**Affiliations:** 1 School of Management, Hefei University of Technology, Hefei 230009, China; 2 Anhui Public Inspection Institute Co., Ltd., Hefei 230051, China; yuandogjie@sina.com; 3 College of Resources and Environment, Anhui Agricultural University, No. 130 Chang jiang West Road, Hefei 230036, China; dandanpan@ahau.edu.cn

**Keywords:** 1,3,6,8-tetrabromocarbazole, 3-bromocarbazole, human serum albumin, spectroscopic method, site II (subdomain IIIA)

## Abstract

The 1,3,6,8-tetrabromocarbazole and 3-bromocarbazole have attracted great attention in the ecotoxicology field recently as hazardous environmental contaminants. In this study, the quenching mechanism of these two substances binding with human serum albumin (HSA) has been investigated with spectroscopic methods. Through fluorescence quenching and binding site experiments with steady-state fluorescence and UV-Vis spectra, the intrinsic fluorescence of HSA quenched by 1,3,6,8-tetrabromocarbazole and 3-bromocarbazole both in static process, are activated by binding to site II (subdomain IIIA) of the HSA. In addition, it was not only found that the conformation and secondary structure of the proteins changes, but also that their spontaneous binding processes were driven by electrostatic interactions as well as hydrophobic forces for HSA-1,3,6,8-tetrabromocarbazole, and by typical hydrophobic forces for HSA-3-bromocarbazole. The above studies are beneficial to enhance our understanding of the ecotoxicology and environmental behaviors of halogenated carbazoles.

## 1. Introduction

Halogenated carbazoles are heteroaromatic compounds with bio-accumulative, potentially persistent, biomagnification and dioxin-like toxicity characteristics, some of which are regarded as hazardous environmental contaminants [1,2]. Up until now, the major causes of emerging halogenated carbazoles have been wastewater discharge and industrial waste in the environment. Although the original sources of these halogenated carbazoles are not well investigated, Parette et al. reported that halogenated indigo dyes were considered as a likely provenance of 1,3,6,8-tetrabromocarbazole, as well as some other halogenated carbazoles [3]. In addition, Zhu et al. identified the chemical structure of 1,3,6,8-tetrabromocarbazole and found 3-bromocarbazole in sediment from Lake Michigan in the United States by using gas chromatographic mass spectrometric techniques [4,5]. According to the physical and chemical properties of halogenated carbazoles identified in the literature, 1,3,6,8-tetrabromocarbazole (log *Kow* = 6.79) and 3-bromocarbazole (log *Kow* = 4.12) chemicals with low aqueous solubilities are not easily to degradable [1]. Therefore, they have received extensive attention because of the environmental problems they cause.

As a multi-purpose macromolecule, human serum albumin (HSA) contains 585 amino acid residues and regulates blood pressure in the body’s circulatory system, which is equipped with transportation and disposition functions relating to exogenous and endogenous compounds such as drugs, theophylline, herbicides, alkaloids, fatty acids, and others [6,7,8,9]. From the protein crystallography, it has been determined that HSA has six subdomains, viz IA, IB, IIA, IIB, IIIA, IIIB, and also three major binding sites, classified as I (subdomain IIA), II (subdomain IIIA) and III, respectively [10,11,12]. It has been shown in the literature that the different types of ligands binding to HSA have a prominent effect on toxicity, metabolism, distribution, absorption and excretion processes, leading to changes in the structure and physiological functions of HSA [13]. Moreover, HSA has been widely used and investigated in many fields, including physicochemical, biochemical, biophysical studies [14,15]. To date, there have been many studies that describe the interaction between HSA and environmental pollutants and HSA, such as perfluorododecanoic acid, heavy metal pollutant-cadmium (II), and triclosan, but none have covered the interaction between HSA and 1,3,6,8-tetrabromocarbazole/3-bromocarbazole with the help of multi-spectroscopic methods such as UV-Vis absorption spectroscopy, fluorescence spectroscopy, circular dichroism (CD) spectroscopy and infrared spectroscopy [16,17,18,19,20].

In the present work, the physicochemical characterizations of 1,3,6,8-tetrabromocarbazole and 3-bromocarbazole binding on HSA are further explored after an analysis of the quenching mechanism, thermodynamic parameters, conformation changes and the binding sites between 1,3,6,8-tetrabromocarbazole or 3-bromocarbazole binding to HSA. This investigation provides detailed information on halogenated carbazoles which will facilitate future understanding of their the ecotoxicological effects, effects on metabolism in vivo, and other environmental behaviors.

## 2. Experimental Section

### 2.1. Materials

Human serum albumin (96%, HSA), flufenamic acid (97%, FA), and phenylbutazone (99%, PB) were purchased from J & K (Beijing, China). The HSA storage solution was dissolved in Tris-HCl buffer (10 mM, pH = 7.4) and placed in a refrigerator at 4 °C in the dark. Digitoxin (≥92%, Dig) was obtained from Sigma-Aldrich (St. Louis, MO, USA). 3-bromocarbazole and 1,3,6,8-tetrabromocarbazole were obtained from Energy Chemical (Shanghai, China), and TCI Development Co., Ltd. (Shanghai, China), respectively. Both were dissolved in anhydrous ethanol. All the other reagents were bought from different chemical companies. Deionized water used in the experiment was obtained from a Milli-Q Plus System (Billerica, MA, USA), and pH values were obtained with a REX PHS-25 digital pH meter (Shanghai, China).

### 2.2. Fluorescence Spectrum

The steady-state fluorescence spectra were measured on a Cary Eclipse fluorescence spectrophotometer (Agilent Technologies) with a 1 cm quartz dish. The experimental temperatures of 298 K, 303 K, and 308 K were controlled by semiconductor temperature control accessories. The excitation and emission slit widths were set at 5 nm, and the scanning speed was 600 nm·min^−1^. To obtain the fluorescence spectra of the tryptophan (Trp) and tyrosine residues (Tyr) in HSA, the excitation wavelength was set at 280 nm and the emission wavelength was set at 290–500 nm with continuous stirring of the solution of HSA-contaminants (1,3,6,8-tetrabromocarbazole and 3-bromocarbazole). Finally, the concentration of the contaminant was changed from 0.1 × 10^−6^ M to 0.8 × 10^−6^ M. In addition, the Tris-HCl buffer solution was scanned in the presence of pollutants, and the corresponding fluorescence signals of only the contaminants were subtracted in order to avoid interference with the fluorescence of the pollutants. In the site marker competition experiments, the concentration of HSA and the three typical site markers (PB, FA and Dig) were fixed at 0.1 × 10^−6^ M, each of which was added before adding the contaminants. The above experiment was repeated three times and an error analysis was carried out.

### 2.3. UV-Vis Absorbance Spectrum

When the scanning ranged from 200–500 nm and the scanning was 300 nm·min^−1^, the UV-Vis absorbance spectra of 1,3,6,8-tetrabromocarbazole and 3-bromocarbazole with HSA were tested on a UV-1800 UV spectrophotometer (Shimadzu, Japan). In each experiment, 1,3,6,8-tetrabromocarbazole and 3-bromocarbazole were gradually added with a gradient of 0.1 × 10^−6^ M to double-sided transparent quartz cuvettes until reaching a final concentration of 0.8 × 10^−6^ M. Finally, the measured data of HSA-1,3,6,8-tetrabromocarbazole and HSA-3-bromocarbazole were deducted from the UV-Vis absorption of only 1,3,6,8-tetrabromocarbazole and 3-bromocarbazole, which were self-generated in the buffer solution.

### 2.4. Synchronous and 3D Fluorescence Spectra Measurements

With a 1 cm path length and 3 mL volume in a quartz cuvette, synchronous fluorescence spectra and three-dimension (3D) fluorescence spectra were measured with a Cary Eclipse fluorescence spectrophotometer (Agilent Technologies, Santa Clara, CA, USA). For Synchronous fluorescence spectra experiments, with parameters of Δλ = 15 nm or 60 nm, the scanning ranges 260–310 nm and 250–320 nm, respectively. For 3D fluorescence spectra measurements, the scanning speed was 9600 nm·min^−1^, the slit of E_x_ and E_m_ were both 10, the excitation wavelength started at 200 nm, and the scanning range of the emission wavelength was 200–450 nm.

### 2.5. CD Spectra

For the CD spectra of HSA and the two environmental pollutants (1,3,6,8-tetrabromocarbazole and 3-bromocarbazol), the scanning wavelength was from 185–260 nm, the step resolution was 0.1 nm, and the scanning speed was 50 nm·min^−1^. Each sample was scanned five times at 298 K and the average values were obtained. Before utilizing a Jasco 810 spectropolarimeter (Tokyo, Japan) that could be used to record the CD spectra, the equipment was first blown with 99.9% of dry nitrogen. The effects of the buffer solution and of the 1,3,6,8-tetrabromocarbazole and 3-bromocarbazole were deducted with all CD spectra, and the change of the HSA secondary structure was measured with CDSSTR software, as supported by the CDPro software package [21].

### 2.6. F.T-IR Spectrum Experiments

The Thermo Scientific Nicolet iS50 FTIR Spectrometer (Waltham, MA, USA) was applied to perform FT-IR spectra analysis of HSA binding to 1,3,6,8-tetrabromocarbazole and 3-bromocarbazole, which measured took the method of attenuated total reflection (ATR) by recording the data taken by 32 scans at 4 cm^−1^ resolution from 500–4000 cm^−1^. Using the OMNIC software, all experimental data reduced the absorbances contributed by 1,3,6,8-tetrabromocarbazole and 3-bromocarbazole in the buffer solution.

## 3. Results and Discussion

### 3.1. Fluorescence Quenching Mechanisms of 1,3,6,8-Tetrabromocarbazole and 3-Bromocarbazole Binding to HSA

**Figure 1:**
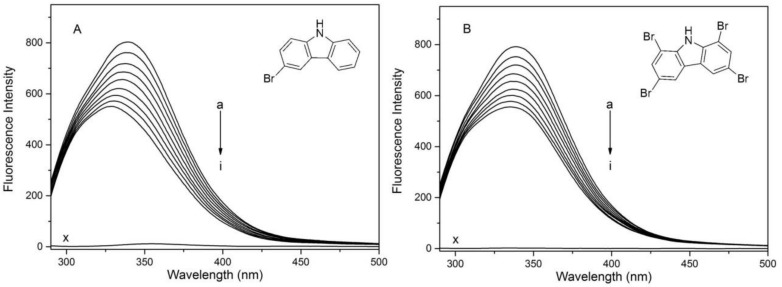
Fluorescence quenching spectra of human serum albumin (HSA) in the presence of different concentrations of 1,3,6,8-tetrabromocarbazole and 3-bromocarbazole. (**A**): HSA-3-bromocarbazole; (**B**): 1,3,6,8-tetrabromocarbazole; λ_ex_ = 280 nm. C_HSA_ = 2 × 10^−6^ M, x: Only 1,3,6,8-tetrabromocarbazole and 3-bromocarbazole concentrations; (a–i) 1,3,6,8-tetrabromocarbazole or 3-bromocarbazole concentrations were 0, 0.1, 0.2, 0.3, 0.4, 0.5, 0.6, 0.7 and 0.8 × 10^−6^ M, respectively.

It is well known that there are many kinds of amino acids in HSA, but among those of which are mainly responsible for producing intrinsic fluorescence of the macromolecule are one tryptophan residue and 18 tyrosine residues measured at 280 nm [22]. When contrasted with the former two, the intrinsic fluorescence of phenylalanine is negligible because of its low quantum yield [23]. As shown in Figure 1, 1,3,6,8-tetrabromocarbazole and 3-bromocarbazole can eliminate the intrinsic fluorescence of HSA, showing that fluorescence at the peak decreases with the addition of these two substances. It was shown that 1,3,6,8-tetrabromocarbazole and 3-bromocarbazole interacted with HSA.

Fluorescence quenching can be divided into two types–static quenching and dynamic quenching. Static quenching usually forms the chromophore—quencher complex, while dynamic quenching is mainly controlled by collision reaction. Mechanisms of fluorescence reaction can be judged by the dependence of temperature or viscosity and are analyzed by the Stern-Volmer equation [23,24,25]:

(1) $$F_{0}/{F=1+K}_{q}\tau_{0}\left[Q\right]{=1+K}_{\mathrm{SV}}\left[Q\right]$$

where *F*_0_ and *F* are the fluorescence intensities before and after the quencher, respectively. The quenching rate constant is *Kq* and the Stern-Volmer quenching constant is *K_SV_*. [*Q*] is the quencher concentration. τ_0_ is the average lifetime of the fluorophore in lack of quencher, which is assumed to be 10^−8^ s for most biomolecules [26,27].

**Figure 2:**
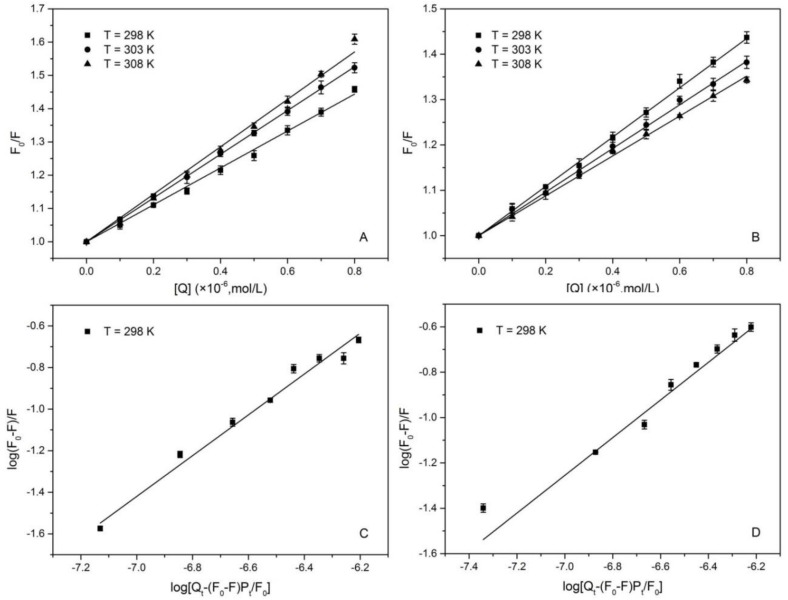
The plots of Stern-Volmer. (**A**): HSA-3-bromocarbazol; (**B**): HSA-1,3,6,8-tetrabromocarbazole) at 298, 303, 308 K and${\;\log[(F}_{0}-F)/F]$ versus $\log\left[Q_{t}{-(F}_{0}{-F)P}_{t}{/F}_{0}\right]$ of HSA-3-bromocarbazole and HSA-1,3,6,8-tetrabromocarbazole systems; (**C**): HSA-3-bromocarbazol; (**D**): HSA-1,3,6,8-tetrabromocarbazole) at 298 K. Data are mean ± SE (bars) (*n* = 3).

A linear plot of *F_0_/F* versus [*Q*] is displayed in Figure 2, and the values of *K_SV_* and *Kq* are listed in Table 1. The linear plots of *F*_0_/*F* versus [*Q*] suggested that there is only one quenching mechanism for HSA-1,3,6,8-tetrabromocarbazole or HSA-3-bromocarbazole systems. Although the value of *K_SV_* for the HSA-3-bromocarbazole complex is proportionally associated with temperature (from 298 K to 308 K), *Kq* is as much as 1000 times larger than the limiting diffusion rate constant (*Kq* is near 1 × 10^10^ M^−1^ s^−1^) of various quenchers with biomolecules. These analysis results imply that the quenching mechanism of the forming HSA-3-bromocarbazole complex was probably a static mechanism. For the HSA-1,3,6,8-tetrabromocarbazole system, *K_SV_* is inversely related with temperature (from 298 to 308 K), whose quenching mechanism is the same as that of the HSA-3-bromocarbazole system, namely a static quenching process [28].

**Figure 3:**
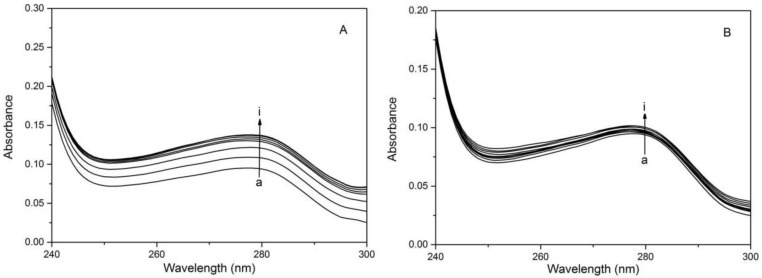
Absorption spectra of HSA with and without 1,3,6,8-tetrabromocarbazole or 3-bromocarbazole. (**A**): HSA-3-bromocarbazole; (**B**): HSA-1,3,6,8-tetrabromocarbazole), C_HSA_ = 2 × 10^−6^ M; (a–i) 1,3,6,8-tetrabromocarbazole or 3-bromocarbazole concentrations were 0, 0.1, 0.2, 0.3, 0.4, 0.5, 0.6, 0.7 and 0.8 × 10^−6^ M, respectively.

Because the absorption peak of HSA at around 280 nm can be caused by the three amino acids (phenylalanine, tyrosine, tryptophan), so the tendency of combining HSA with 1,3,6,8-tetrabromocarbazole or 3-bromocarbazole was judged as either static or dynamic quenching by using UV-visible spectroscopy [23,29]. Static quenching results in some variations of the absorption spectrum in the fluorophore excited states, while the quenching does not change. From Figure 3, it is obvious that the combination of these two substances with HSA forms a complex, which can be seen through the phenomenon that the UV absorption peak at 280 nm changes with the addition of these two substances. This indicates that the quenching mechanism cause the interaction of the interacting HSA with 1,3,6,8-tetrabromocarbazole or 3-bromocarbazole is indeed static quenching, which is in good agreement with the discussion about *K_SV_*.

### 3.2. Binding Constants and Location

Small molecules interacted with a series of equivalent sites in a biomolecule which is independent, and clear binding sites between HSA and 1,3,6,8-tetrabromocarbazole or 3-bromocarbazole can be identified using double-logarithmic formula [30]:

(2) $${\log[(F}_{0}-F)/{F]=\mathrm{logK}}_{a}+n\;\log\left[Q_{t}{-(F}_{0}{-F)P}_{t}{/F}_{0}\right]$$

where the meaning of *F_0_*, and *F* are consistent with that of Equation (1), [*Qt*] is the total quencher concentration, [*Pt*] is the total protein concentration, *Ka* is the binding affinity constant, and *n* is the average number of binding sites. The ${\log[(F}_{0}-F)/F]$ versus the $\log\left[Q_{t}{-(F}_{0}{-F)P}_{t}{/F}_{0}\right]$ of the HSA-3-bromocarbazole and HSA-1,3,6,8-tetrabromocarbazole systems is shown in Figure 3, the binding affinity constant and binding number n were listed in Table 2. Table 2 show that the values of *n* were almost equal to 1, indicating that there is only one site at which 1,3,6,8-tetrabromocarbazole or 3-bromocarbazole interacted with HSA at different temperatures. Hence, the marker-site competition experiments were designed to determine which of the three sites existed in HSA. The three binding sites are PB for site I (subdomain IIA), FA for II (subdomain IIIA) and Dig for III [31,32]. By comparing the binding constants of HSA-1,3,6,8-tetrabromocarbazole and HSA-3-bromocarbazole systems in Table 3, *Ka* has changed dramatically after the addition of FA, which reveals that site II (subdomain IIIA) is the main binding site for HSA binding to these two substances.

### 3.3. Thermodynamic Parameters and Interaction Modes

The binding force between small molecules and their binding targets are made up of diversified non-covalent interactions, such as electrostatic interactions, Van der Waals interactions, hydrophobic forces, salt bridges, π effects and steric contacts within the binding site [33,34]. Free energy change *ΔG*, entropy change *ΔS* and enthalpy change *ΔH* belong to the thermodynamic parameters, which were calculated by Van’t Hoff formulas (3) and (4) to decide the interaction modes.

(3) $$\ln K_{a}=-∆H/RT+∆S/R$$

(4)     ∆G=∆H−T∆S

where *K_a_* is the binding constant at the different temperatures, and the gas constant *R* is about 8.314 J·mol^−1^·K^−1^. From Table 2, the negative signs of the *ΔG* value both in HSA-1,3,6,8-tetrabromocarbazole and HSA-3-bromocarbazole systems, indicated that a spontaneous process occurred. According to Ross’s theory, the positive signs of the *ΔH* and *ΔS* values indicated that 3-bromocarbazole bound to HSA depending on typical hydrophobic forces with endothermic performance, and the negative signs of *ΔH* and positive signs of *ΔS* for 1,3,6,8-tetrabromocarbazole interacted with HSA, driven by electrostatic interactions and hydrophobic forces with exothermic performance. Both are spontaneous processes [33,35].

### 3.4. Study of Conformational Changes of HSA

In order to investigate what happened on the conformation changes of HAS in the presence of these two pollutants, CD spectra, FT-IR spectra, synchronous fluorescence measurements and 3D fluorescence spectra were measured.

**Figure 4:**
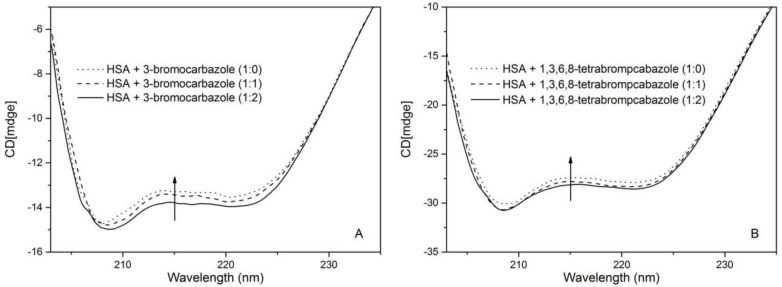
Absorption spectra of HSA with and without 1,3,6,8-tetrabromocarbazole or 3-bromocarbazole. (**A**): HSA-3-bromocarbazole; (**B**): HSA-1,3,6,8-tetrabromocarbazole), C_HSA_ = 2 × 10^−6^ M; (a–i) 1,3,6,8-tetrabromocarbazole or 3-bromocarbazole concentrations were 0, 0.1, 0.2, 0.3, 0.4, 0.5, 0.6, 0.7 and 0.8 × 10^−6^ M, respectively.

The CD spectroscopy is a powerful tool for analyzing the conformation of biological macromolecules, which have two negative bands at 208 (π→π*) and 220 nm (n→π*) in Figure 4. *α*-helices, β-sheets, β-turns, and random coils were calculated to depict the HSA secondary structure and these results are listed in Table 4. A characteristic shape was shown in the CD spectrum of HSA in presence of 1,3,6,8-tetrabromocarbazole or 3-bromocarbazole is consistent with the a-helix-rich secondary structure [36,37,38]. As shown in Table 5, the *α*-helical content of only HSA was decreased from 48.8% to 34.1%, and β-sheets content increased from 20.7% to 33.7% upon its addition to 1,3,6,8-tetrabromocarbazole. And the a-helical content of HSA decreased from 48.8% to 34.1% and β-sheets content increased from 20.7% to 33.7% upon its addition to 3-bromocarbazole. The *α*-helix value decreasing and β-sheets content value indicated that the interaction of HSA with these two pollutants contributed to a partial protein unfolding and its orderly secondary structure changed.

**Figure 5:**
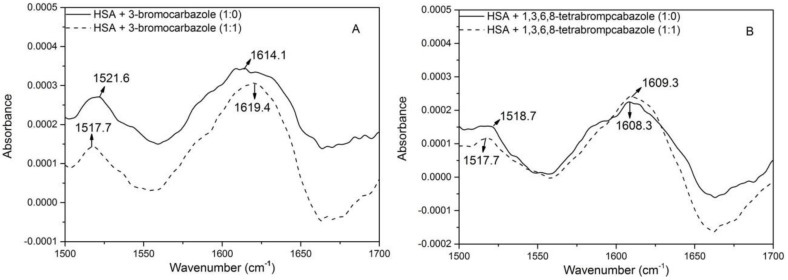
FTIR spectra (**A**): HSA-3-bromocarbazol; (**B**): HSA-1,3,6,8-tetrabromocarbazole): C_HSA_ = 2.0 × 10^−6^ M, and the molar ratios of HSA-3-bromocarbazol and HSA-1,3,6,8-tetrabromocarbazole systems were 1:0, 1:1, and 1:2, respectively.

The FTIR spectra for HSA acquired over a series of 1700–1500 cm^−1^ showed the secondary structure of HSA is related to the amide I and amide II bands, which were located at around 1600–1700 cm^−1^ and 1500–1600 cm^−1^, respectively. The amide II band is a C–N stretch coupled with a N–H bending mode the amide I band and the amide II bands are mainly associated with the C=O stretch band. When analyzing the frequencies of the peptide moiety of these two binds, the secondary structure of the HSA change can be realized [39]. The FT-IR spectra of HSA binding to 1,3,6,8-tetrabromocarbazole or 3-bromocarbazole were displayed in Figure 5. The amide I band peak position in HSA-3-bromocarbazole and HSA-1,3,6,8-tetrabromocarbazole systems red moved from 1614.1 cm^−1^ to 1619.4 cm^−1^, and from 1608.3 cm^−1^ to 1609.3 cm^−1^ respectively, and that of the amide II band blue shift from 1521.6 cm^−1^ to 1517.7 cm^−1^and from 1518.7 cm^−1^ to 1517.7 cm^−1^, respectively. The positions and shapes of the peaks changed, resulting in the rearrangement of the polypeptide carbonyl hydrogen-bonding network and conformational changes when 1,3,6,8-tetrabromocarbazole and 3-bromocarbazole interacted with HSA [40]. Moreover, the change of the amide I band intensity is caused by the content of the protein α-helical structure, confirming that the structure of the HSA changed [7].

**Figure 6:**
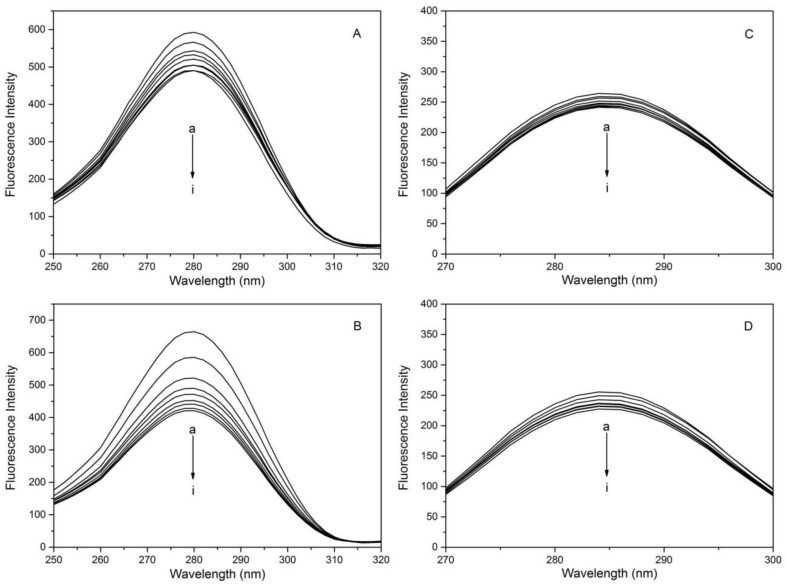
Synchronous fluorescence spectra of HSA in the presence of different concentration of 1,3,6,8-tetrabromocarbazole or 3-bromocarbazole at 298 K: *Δλ* = 60 nm. (**A**): HSA-3-bromocarbazol; (**B**): HSA-1,3,6,8-tetrabromocarbazole) and *Δλ* = 15 nm; (**C**): HSA-3-bromocarbazol; (**D**): HSA-1,3,6,8-tetrabromocarbazole), C_HSA_ = 2 × 10^−6^ M; (a–i) 1,3,6,8-tetrabromocarbazole or 3-bromocarbazole concentrations at 0, 0.1, 0.2, 0.3, 0.4, 0.5, 0.6, 0.7 and 0.8 × 10^−^^6^ M, respectively.

When adjusting the emission and excitation wavelengths at 15 and 60, synchronous fluorescence spectra can be observed with the microenvironment changing around the amino acids of HSA, such as Trp and Tyr [41,42]. For HSA-1,3,6,8-tetrabromocarbazole or 3-bromocarbazole systems, Figure 6 showed that change in fluorescence quenching intensity of Tyr when Δλ = 15 was weaker compared to that of Trp when Δλ = 60, suggesting that Trp contributes more to quenching the intrinsic fluorescence of HSA in the excitation wavelength of 280 nm.

**Figure 7:**
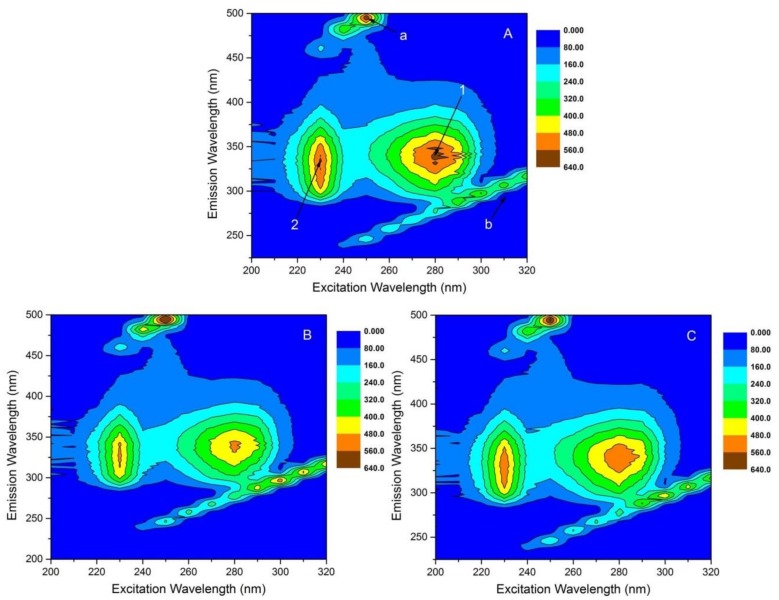
3D fluorescence spectra of HSA in the absence and presence of 1,3,6,8-tetrabromocarbazole or 3-bromocarbazole at 298 K. C_HSA_ = 2 × 10^−6^ M; (**A**): Only HSA, 1:0; (**B**): HSA-3-bromocarbazole, 1:1; (**C**): HSA-1,3,6,8-tetrabromocarbazole, 1:1.

A 3D fluorescence spectrum can provide abundant spectral information such as the relative strength of the fluorescence peak and the fluorescence peak position, which can better express the original 3D fluorescence spectrum property of the sample. Therefore, detailed analysis of HSA structural changes caused by the addition of 1,3,6,8-tetrabromocarbazole or 3-bromocarbazole by 3D fluorescence spectra is helpful to fully understand the conformational interference from these two pollutants. The characteristic parameters of 3D fluorescence are summarized in Table 5 and the corresponding contour map data were shown in Figure 7. The Peak a (λ_ex_ = λ_em_) is the second-order scattering, and the other peak b (2λ_ex_ = λ_em_) the Rayleigh scattering [43,44]. In addition, the strong Peaks 1 illustrated Trp and Tyr residues on proteins and Peaks 2 illustrated the spectral features of the polypeptide backbone structure on proteins. Their fluorescence intensities decreased with the addition of 1,3,6,8-tetrabromocarbazole or 3-bromocarbazole, suggesting that some micro-environmental and conformational changes occurred upon the binding of 1,3,6,8-tetrabromocarbazole or 3-bromocarbazole to HSA,and these results are in agreement with analysis obtained from CD and FT-IR spectra [45,46].

### 3.5. Fluorescence Resonance Energy Transfer

**Figure 8:**
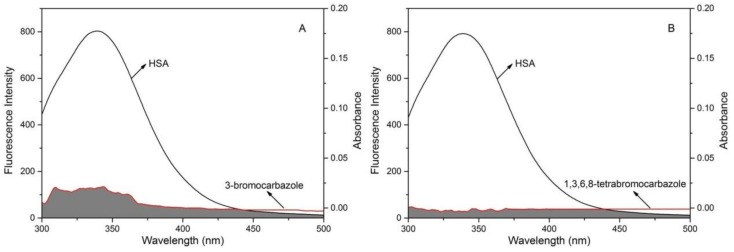
The overlaps of HSA fluorescence spectra and 1,3,6,8-tetrabromocarbazole or 3-bromocarbazole absorption spectra. (**A**): HSA-3-bromocarbazole; (**B**): HSA-1,3,6,8-tetrabromocarbazole.

As a non-destructive spectroscopy based method, the fluorescence resonance energy transfer (FRET) is extensively applied to determine the molecular distances between the donor and the acceptor molecules. Only the donor molecule’s emission spectrum overlaps with the acceptor molecule’s absorption spectrum is reasonable [44,47,48,49]. The distance (r) between the donor HSA and the acceptor 1,3,6,8-tetrabromocarbazole or 3-bromocarbazole molecules can be expressed through the theory of the above methods, which is Förster’s nonradiation energy theory. The efficiency of energy transfer (EFRET) can be calculated as:

(5) $$E=1-\frac{F}{F_{0}}=\frac{R_{0}{}^{6}}{R_{0}{}^{6}+r^{6}}$$

where *F*_0_ and *F* are the fluorescence intensities of HSA in the absence and presence of 1,3,6,8-tetrabromocarbazole or 3-bromocarbazole; the distance *r* and *R*_0_ with 50% is the energy transfer efficiency between donor and acceptor molecules, and. *R*_0_ can be acquired as:

(6) $$R_{0}{}^{6}=8.8\times 10^{-25}K^{2}N^{-4}\phi \;J$$

where *K*^2^ is the factor related to the geometry of the donor and acceptor dipoles, *N* is the refractive index of a medium, *ϕ* is the donor fluorescence quantum yield, and *J* is the overlap integral of the donor emission. The acceptor absorption spectra can be calculated from the following equation:

(7) $$J=\frac{\sum^{\text{​}}F(\lambda )\varepsilon \left(\lambda \right)\lambda^{4}∆\lambda }{\sum^{\text{​}}F\left(\lambda \right)∆\lambda }$$

where *F(λ)* is the fluorescence intensity of the donor ranging from *λ* to (*λ*+Δ*λ*) and *ε(λ)* is the molar absorption coefficient of the acceptor at wavelength *λ*, and *K*^2^, *N* and *ϕ* were taken as 2/3, 1.361 and 0.14, respectively. For HSA-1,3,6,8-tetrabromocarbazole and HSA-3-bromocarbazole systems, the donor molecule’s emission spectrum overlaps with the acceptor molecule’s absorption spectrum are as shown in Figure 8, and the FRET parameters of aforementioned equations are obtained in Table 6. The binding distances of *r* is 2.23 nm and 2.27 nm, respectively. Both are less than 8 nm, and 0.5*R_0_* < *r* < 1.5*R_0_*, suggesting the high probability for the occurrence of an energy transfer process from the intrinsic fluorophore donor of HSA to 1,3,6,8-tetrabromocarbazole and 3-bromocarbazole [50].

## 4. Conclusions

The quenching mechanisms of 1,3,6,8-tetrabromocarbazole and 3-bromocarbazole binding to HSA were both static processes by steady-state fluorescence, which was confirmed by UV-Vis spectra. The binding site titration experiments suggest that these two substances interacted with HSA at site II (subdomain IIIA), with energy transfer performance and description of thermodynamic parameters indicating that the spontaneous binding between HSA and 1,3,6,8-tetrabromocarbazole or 3-bromocarbazole both depends on electrostatic interactions as well as hydrophobic forces, and typical hydrophobic forces. In addition, what we can acquire learn from this study is that 1,3,6,8-tetrabromocarbazole and 3-bromocarbazole binding to HSA results in protein unfolding revealed by CD spectroscopy, the rearrangement of the polypeptide carbonyl hydrogen-bonding network revealed by FTIR spectra and microenvironment and conformational changes revealed by 3D fluorescence. These results show that the protein structure changes induced by 1,3,6,8-tetrabromocarbazole and 3-bromocarbazole are potentially harmful to the human body. Moreover, it is helpful to fully understand the metabolic behavior of other halogenated carbazoles after acting on the human body, and the toxicity mechanism between the human body and these compounds.

## Figures and Tables

**Table 1:** Stern-Volmer quenching constants for the interactions of 3-bromocarbazole and 1,3,6,8-tetrabromocarbazole with HSA at different temperatures.

System	*T* (K)	*K_SV_* ± *SD ^b^* (× 10^5^·M^−1^)	*Kq* (× 10^13^·M^−^^1^·s^−1^)	*R* ^2^
	298	5.55 ± 0.02	5.55	0.998
HSA-3-bromocarbazole	303	6.57 ± 0.01	6.57	0.999
	308	7.13 ± 0.01	7.13	0.997
	298	5.43 ± 0.03	5.43	0.999
HSA-1,3,6,8-tetrabromocarbazole	303	4.81 ± 0.04	4.81	0.998
	308	4.40 ± 0.03	4.40	0.999

*R*^2^ is the correlation coefficient. *SD ^b^* is the standard deviation of the *K_SV_* values.

**Table 2:** Binding constants and thermodynamic parameters of HSA-3-bromocarbazole and HSA-1,3,6,8-tetrabromocarbazole binding systems.

Compound	T(K)	*Ka ± SD*^b^ (×10^5^·M^−1^)	*n*	*R* *^2^*	Δ*H* (kJ·mol^−1^)	Δ*G* (kJ·mol^−1^)	Δ*S* (J·mol^−1·^K^−1^)
HSA-3-bromocarbazole	298	2.18 ± 0.39	0.956	0.978		−30.49	
	303	2.67 ± 0.15	0.847	0.988	24.65	−31.41	185.02
	308	3.01 ± 0.20	0.78	0.984		−32.33	
HAS-1,3,6,8-tetrabromocarbazole	298	3.39 ± 0.40	0.703	0.948		−31.57	
	303	3.05 ± 0.14	0.866	0.993	−19.06	−31.78	41.96
	308	2.64 ± 0.14	0.870	0.993		−31.99	

*R^2^* is the correlation coefficient. *SD* ^b^ is the standard deviation of *n* values.

**Table 3:** Effects of the site probes on the binding constants of 3-bromocarbazole and 1,3,6,8-tetrabromocarbazole to HSA.

System	Site Marker	*K*^a^*± SD*^b^ (×10^5^ M^−1^)	R^2^
HSA-3-bromocarbazole	Blank	2.18 ± 0.39	0.978
	PB	1.92 ± 0.15	0.993
	FA	1.15 ± 0.17	0.983
	Dig	1.62 ± 0.26	0.976
HSA-1,3,6,8-tetrabromocarbazole	Blank	3.39 ± 0.40	0.948
	PB	3.31 ± 0.19	0.989
	FA	2.78 ± 0.10	0.999
	Dig	3.12 ± 0.33	0.997

*R^2^* is the correlation coefficient; *SD* ^b^ is the standard deviation for the *K*^a^ value.

**Table 4:** Conformation changes in the secondary structure of HSA with and without 3-bromocarbazole and 1,3,6,8-tetrabromocarbazole.

Sample	Secondary Structure (%)			
	α-Helix	β-Sheet	β-Turn	Random Coil
Only HSA	48.8	20.7	15.2	16.0
HSA + 3-bromocarbazole (1:1)	42.0	26.7	15.7	15.7
HSA + 3-bromocarbazole (1:2)	34.1	33.7	16.9	15.3
HSA + 1,3,6,8-tetrabromocarbazole (1:1)	41.8	26.8	14.9	16.6
HSA + 1,3,6,8-tetrabromocarbazole (1:2)	38.9	27.8	16.6	16.7

**Table 5:** Characteristic parameters of Peak 1 and Peak 2.

Compound	Peaks	Peaks Position λ_ex_/λ_e__m_ (nm/nm)	Stokes Δλ (nm)	Intensity
Only HSA	Peak 1	280.0/348.0	68.0	619.2
	Peak 2	230.0/334.0	104.0	575.7
HSA-3-bromocarbazole	Peak 1	280.0/338.0	58.0	499.8
	Peak 2	230.0/328.0	98.0	505.5
HSA-1,3,6,8-tetrabromocarbazole	Peak 1	280.0/338.0	62.0	545.3
	Peak 2	230.0/330.0	100.0	553.2

**Table 6:** Parameters of *E*, *J*, *R_0_* and *r* of HSA-3-bromocarbazole and HSA-1,3,6,8-tetrabromocarbazole systems.

Compound	*E* (%)	J (cm^3^·L·mol^−1^)	*R_0_*(nm)	*r* (nm)
HSA-3-bromocarbazole	9.96	1.04×10^−14^	2.34	2.23
HSA-1,3,6,8-tetrabromocarbazole	7.55	0.90×10^−14^	2.28	2.27
